# Supplementary material for: Biocytin-Labeling in Whole-Cell Recording: Electrophysiological and Morphological Properties of Pyramidal Neurons in CYLD-Deficient Mice
Source: Molecules. 2023 May 15;28(10):4092. doi: 10.3390/molecules28104092 (PMC10221440; doi:10.3390/molecules28104092)
Supplement: Supplementary file 1 [file molecules-28-04092-s001.zip › molecules-2344711-supplementary.pdf]

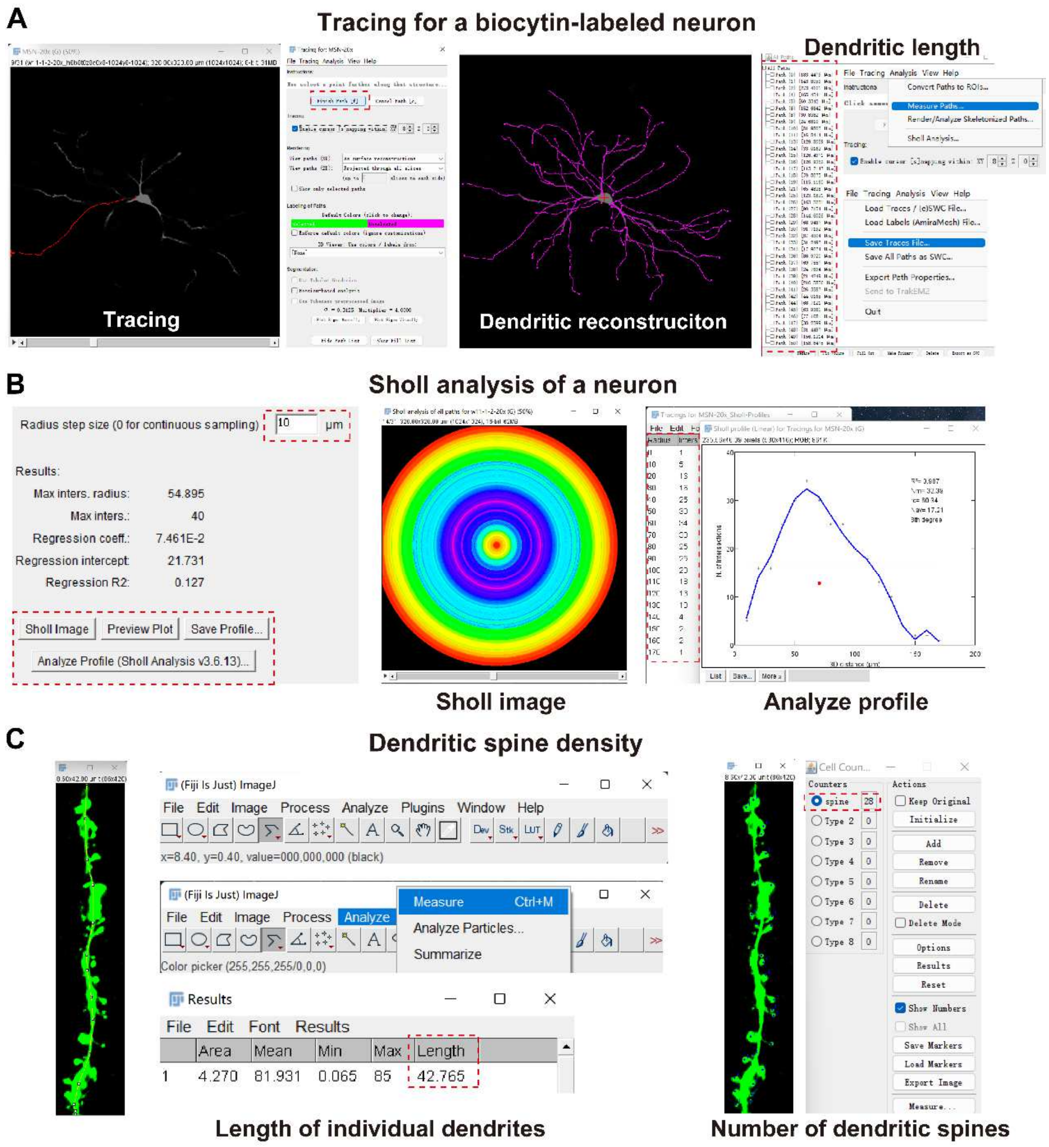

Figure S1 Illustration of ImageJ analysis of biocytin labeled neurons.

(A) Tracing of a biocytin-loaded neuron, and analysis of dendritic length with "Simple Neurite Tracer" in ImageJ. (B) A manual reconstruction of Sholl analysis. (Left) Radius step size is set to 10 µm. (Middle) Sholl image. (Right) The number of dendrite intersections in the region 10-170 µm from the cell soma. (C) Testing the length of a single dendrite and number of dendritic spines. The spine density of individual dendrites was calculated as the number of spines per unit length.
